# Berberine promotes primordial follicle activation and increases ovulated oocyte quantity in aged mice

**DOI:** 10.1186/s10020-024-01042-z

**Published:** 2024-12-20

**Authors:** Shuang Liu, Weiyong Wang, Huiyu Liu, Hongwei Wei, Yashuang Weng, Wenjun Zhou, Xiaodan Zhang, Sihui He, Ye Chen, Yahong Wang, Meijia Zhang, Xin Chen

**Affiliations:** 1 The Innovation Centre of Ministry of Education for Development and Diseases, School of Medicine, South China University of Technology, Guangzhou, 510006 China; 2 Reproductive Medicine Center, Shunde Hospital of Southern Medical University (The First People’s Hospital of Shunde), Foshan, 528300 China

**Keywords:** Berberine, Primordial follicle activation, Akt, Premature ovarian insufficiency

## Abstract

**Background:**

Primordial follicle activation is vital for the reproduction of women with advanced age and premature ovarian insufficiency (POI). But there is a lack of effective and safe therapeutic options to activate their primordial follicles in vivo. Berberine (BBR) possesses multiple pharmacological properties, but its impact on primordial follicle activation remains unclear.

**Methods:**

The role of BBR on primordial activation was investigated by neonatal mouse ovary culture and intraperitoneal injection, and by human ovarian fragment culture. Furthermore, the effect of BBR on the quantity of ovulated oocytes was investigated by the intragastric administration of aged mice.

**Results:**

BBR in vitro culture and in vivo intraperitoneal injection significantly increased growing follicle number and phosphorylated protein kinase B (p-Akt) levels in neonatal mouse ovaries. BBR also significantly increased the relative fluorescence intensities of p-Akt in the oocytes of primordial follicles. BBR-increased the number of growing follicles and the levels of p-Akt were blocked by LY294002, an inhibitor of phosphatidylinositol 3-kinase (PI3K). Furthermore, BBR intragastric administration significantly increased the quantity of ovulated oocytes in aged mice. Moreover, BBR significantly increased growing follicle proportion and p-Akt levels in cultured human ovarian fragments.

**Conclusion:**

BBR promotes mouse and human primordial follicle activation through the PI3K/Akt pathway in oocytes, and improves the quantity of ovulated oocytes in aged mice. Our results suggest a potential use of oral medicine BBR to improve fertility in POI patients and aged women.

**Supplementary Information:**

The online version contains supplementary material available at 10.1186/s10020-024-01042-z.

## Introduction

Female mammals establish their primordial follicle pool during the perinatal period to provide fertilizable oocytes throughout the reproductive lifespan (Li et al. 2020). The primordial follicle is distinguished by a relatively dormant oocyte encircled by a layer of flattened pregranulosa cells (Zhang et al. 2023b). The oocyte diameter increases and the flat pregranulosa cells transform into a cubic shape when the primordial follicles are activated (Chen et al. 2020). The mammalian target of rapamycin (mTOR) within pregranulosa cells stimulates the expression of proto-oncogenic receptor tyrosine kinase (KIT) ligand (KITL) (Zhang et al. 2022b). KITL binds to KIT in the oocytes and activates the PI3K/Akt pathway, and then phosphorylated forkhead Box O3a (FOXO3a) is transported out of the oocyte nucleus, resulting in the primordial follicle activation (Zhang et al. 2023b, 2024a). Once the activated primordial follicles enter into the growing stage, they cannot be preserved (Chen et al. 2020). Thus, the ordered activation of primordial follicles is vital for the reproduction lifespan of females. Disarranged primordial follicle activation would lead to premature ovarian insufficiency (POI) (Nash and Davies 2024).

POI could be caused by both the genetic and non-genetic factors. The genetic factors include chromosomal abnormalities and genetic variants (Ishizuka 2021; Ke et al. 2023). Nongenetic factors mainly include iatrogenic factors and autoimmune disorders (Ke et al. 2023; Lang et al. 2024). The incidence of POI is increasing each year, and there is a trend toward younger onset. In most cases, patients with POI have a few of residual primordial follicles that are physically inactivated, and they cannot obtain their genetic offspring using traditional assisted reproductive technology (ART) (Zhang et al. 2021b). Current research on POI treatment mainly includes in vitro activation (IVA), intra-ovarian infusion of platelet-rich plasma (PRP), and stem cell therapy (Huang et al. 2022). However, these strategies are limited to individual cases and are associated with the risks of invasive surgery and a low success rate. Therefore, these treatments are not widely applied in clinical practice (Ding et al. 2023). Aged women also exhibit reduced oocyte quantity and quality, resulting in impaired fertility (Zhang et al. 2020a; Smits et al. 2021). Hence, it needs to develop effective and safe therapeutic strategies to enhance fertility in POI patients and aged women.

Berberine (BBR) is a quaternary ammonium isoquinoline alkaloid extracted from natural herbs, such as *Coptis chinensis* and other *Berberis* plants (Song et al. 2020). As an oral medicine, BBR is usually used to treat intestinal infections caused by bacteria and virus (Yu et al. 2020; Zhang et al. 2021a). BBR exerts several pharmacological effects (Khezri et al. 2024). In vivo, BBR resists lipopolysaccharide (LPS)-stimulated inflammatory responses by downregulating nuclear factor-κB (NF-κB) subunit p65^Lys310^ acetylation in mice, and inhibits cholesterol absorption and decreases plasma low-density lipoprotein cholesterol levels in hamsters (Guo et al. 2023; Zhang et al. 2023a). BBR gavage alleviates ovarian morphological lesions, and improves ovulation in letrozole- and testosterone-induced rat polycystic ovary syndrome (PCOS) models (Wang et al. 2021; Yu et al. 2021). In vitro, BBR promotes the activation of the PI3K/Akt signaling pathway in cultured rat granulosa cells (Yu et al. 2021). Akt activity in oocytes is crucial for primordial follicle activation (Zhang et al. 2024b). Therefore, we explored the influence of BBR on the activation of primordial follicles and its potential mechanisms were also investigated.

## Materials and methods

### Animals and chemicals

ICR mice at adolescent (3 weeks old) and adult (2 and 10 months old) stages were purchased from the Guangdong Medical Laboratory Animal Center (Foshan, China). The animals were raised under controlled conditions, with temperatures maintained at 22-24 ℃, humidity levels ranging between 50 and 70%, and a 12/12 h light/dark cycle. They had unrestricted access to sufficient water and food. The two-month-old mice were mated in a 1:1 ratio to produce neonatal mice, and the day of birth was deemed 0.5 days postpartum (dpp). Three dpp female mice were subjected to in vitro ovary culture or intraperitoneal injection. In the intragastric administration experiment, adolescent and aged female mice were treated with water (control) or water supplemented with BBR (MedChemExpress, New Jersey, USA, HY-N0716). All procedures of the animal experiments were approved by the Animal Care and Use Committee of South China University of Technology (approval number: 2022102, approved on 30 December 2022). All reagents were obtained from Sigma-Aldrich (St. Louis, Missouri, USA) unless otherwise specified. The supplementary Table S1 provides a list of primary antibodies.

### Mouse ovary culture

The ovaries from three dpp female mice were washed in sterile phosphate-buffered saline (PBS) and then were cultured on a Millipore insert (Millipore, Billerica, MA, USA) within a six-well culture plate (NEST, Beijing, China). The culture medium is Dulbecco’s modified Eagle’s medium/Ham’s F12 nutrient mixture (DMEM/F12, Thermo Fisher Scientific, Waltham, MA, USA) and its components have been reported previously (Han et al. 2023). In treatment groups, BBR (0-500 ng/mL) and/or PI3K inhibitor LY294002 (10 µM; MedChemExpress, New Jersey, USA) were supplemented in the medium for designated days. In bromodeoxyuridine (BrdU) incorporation assay, three dpp mouse ovaries were cultured for two days followed by further two-hour incubation with 10 µM BrdU. BBR and LY294002 were prepared in ultrapure water and in dimethyl sulfoxide (DMSO) as stock solution respectively. The same volumes of DMSO (less than 0.1%) were added as control. The ovaries were cultured at 37 °C and 5% CO_2_, and the medium was changed every the other day. Ovaries were harvested for gene and protein detection, immunofluorescence staining, and follicle counting at designated times.

### Mouse intraperitoneal injection and intragastric administration experiments

The injection dosage was based on the concentration selected from in vitro culture experiment, in which the volume ratio (mg/L) was converted to the mass ratio (mg/kg). Three dpp female mice (body weight: 2.58 ± 0.29 g) were intraperitoneally injected with normal saline (control) or 0.05 mg/kg BBR once a day for two serial days. The ovaries were harvested 24 h after the end of injection for immunofluorescence staining and protein detection, and 48 h after the end of injection for follicle counting. The BBR intragastric administration dose was based on the concentration selected from in vitro culture and the bioavailability of BBR (Chen et al. 2011; Behl et al. 2022). Female mice at 3 weeks (adolescent) and 10 months (aged) were intragastrically treated with BBR (0-20 mg/kg/day) for a week. The ovaries were harvested after the last intragastric administration for follicle counting and immunofluorescence staining. After being fed for further three weeks, the aged mice were used for ovary follicle counting and oocyte quantity and quality analyzing.

### Oocyte quantity and quality analysis

The aged mice were super-ovulated by injecting 5 IU of pregnant mare serum gonadotropin (PMSG), followed by 5 IU of human chorionic gonadotropin (HCG) after 48 h (PMSG and HCG were purchased from Ningbo Second Hormone Factory, Zhejiang, China). Thirteen hours later, cumulus-oocyte complexes (COCs) were collected from the oviduct ampulla and treated with 0.1% hyaluronidase to obtain the oocytes. An assessment was carried out on the quantity of oocytes, and their quality was determined by examining the spindle morphology, the levels of reactive oxygen species (ROS), and the mitochondrial membrane potential (ΔΨm). The content of oocyte ROS and ΔΨm were measured according to corresponding kit instructions (Beyotime, Beijing, China).

### Human ovary cortex fragments culture

Ovarian cortical tissues were obtained from five women aged 28-37 years (31.40 ± 3.01 years) while undergoing laparoscopy for endometriosis at Shunde Hospital of Southern Medical University, Foshan, Guangdong, China. An informed written consent was obtained from each patient before surgery. The study was conducted following the Declaration of Helsinki. The human ovarian tissue collection and usage were approved by the Ethics Committee of Shunde Hospital of Southern Medical University (approval number: KYLS20221203, approved on 16 December 2022). The non-pathological part of human ovarian tissue was put in precooled PBS supplemented with penicillin–streptomycin, and was transported to the laboratory immediately.

The ovarian tissues were cut into cubic fragments of approximately 1 mm^3^ under aseptic conditions. Several fragments from each sample were frozen to detect protein expressions or fixed in 4% paraformaldehyde (PFA; Solarbio, Beijing, China) to analyze follicles (uncultured). The remaining fragments were evenly and randomly divided for a six-day culture in medium (control) or a four-day culture in medium supplemented with BBR followed by a two-day culture in BBR-free medium (BBR). The fragments were cultured under the same conditions as mentioned in the mouse ovary culture section. The fragments were collected after a four-day culture to analyze the protein levels or a six-day culture to analyze the follicles.

### Histological and morphological analysis

Ovarian tissue specimens were placed in 4% PFA for overnight fixation and then embedded in paraffin. The paraffinized samples were sliced into 5 μm thick sections using a serial cutting technique, and affixed to glass slides. These slides underwent deparaffinization in xylene, followed by hydration using graded alcohol. To visualize the tissues, hematoxylin staining (Solarbio, Beijing, China) was performed. The classifications of primordial, growing, and atretic follicles were described in previous work (Li et al. 2022). In neonatal mouse ovaries, the total number of primordial follicles was counted in every five section and was calculated by averaging the number of follicles per section × total number of sections. Serial sections were analyzed to count growing and atretic follicles in neonatal, adolescent and aged mice ovaries, as well as human ovarian tissues. Only follicles with visible nuclei were counted to avoid double counting. All sections were evaluated by two individuals for cross-reference.

### Immunofluorescent staining

The slides attached to different groups of ovarian tissue sections were deparaffinized and hydrated, as described above. Antigen retrieval of these sections was performed using 0.01% sodium citrate buffer under 95-98 ℃. After the buffer cooled to room temperature, the sections went through a blocking step with 10% donkey serum for one hour. The sections were incubated with primary antibodies overnight at 4 ℃, and then with corresponding secondary antibodies (Alexa Fluor™ 488- or Alexa Fluor™ 555- conjugated, 1:200, Thermo Fisher Scientific) for one hour at 37 ℃. Following this, 4’,6-diamidino-2-phenylindole (DAPI, 1:200, Beyotime, Beijing, China, C1002) was used to stain the nuclei. These sections were then imaged using a confocal microscope (LSM 800, Carl Zeiss, Oberkochen, Germany). To determine the proportion of granulosa cells and follicles displaying positive signals, and the proportion of FOXO3a nuclear export in oocytes, as well as the cells with positive signals, a total of five largest sections in each ovary were selected for analysis. The proportion was calculated by dividing the number of cells or follicles displaying positive signals by the total number of cells or follicles. The mean values of 15 sections obtained from three ovaries in each experiment was considered as one independent sample data. Moreover, the Zeiss Zen 3.0 software (Carl Zeiss, Oberkochen, Germany) was employed to compute the relative fluorescence intensity. This was completed by dividing the intensity of fluorescence from the cells by the intensity identified in the background. Oocytes collected from aged mice underwent fixation in 4% PFA at room temperature for 30 min and permeabilized in PBS containing 0.1% Triton X-100 for 20 min. After a blocking step with 10% donkey serum at 37 °C for one hour, the oocytes were incubated overnight at 4 °C with Alexa Fluor 488-anti-alpha tubulin antibody (1:400, Abcam, ab197737). The oocytes were washed with PBS supplemented with 0.1% Tween and then subjected to DAPI staining for five minutes. Subsequently, the oocytes were transferred onto slides and photographed using a confocal microscope.

### RNA isolation and quantitative real-time PCR (qRT-PCR)

RNA extraction was performed on six neonatal mouse ovaries per group using ReliaPrep™ RNA Miniprep Systems (Promega, Madison, WI, USA, Z6111). To synthesize cDNA, 1 µg of total RNA from each group was subjected to reverse transcription using the GoScript™ Reverse Transcription System (Promega, Madison, WI, USA, A5001). Data normalization was performed using ribosomal protein L19 (*Rpl19*). The relative expression levels of mRNA were calculated using the 2^−ΔΔCT^ method. The details of the primers can be found in Supplementary Table S2.

### Western blotting analysis

Six neonatal mouse ovaries and human ovarian tissues from each group were used to extract total protein. A total of 20 micrograms of protein from each group were separated by 10% sodium dodecyl sulfate (SDS) -polyacrylamide gels and then transferred to polyvinylidene fluoride (PVDF) membranes (Millipore, MA, USA). The membranes were incubated with 5% skim milk (Absin, Shanghai, China) for one hour followed by an incubation with primary antibodies (Supplementary Table S1) at 4℃ overnight. Next, the membranes underwent an incubation with the secondary antibodies of the corresponding species (anti-mouse or anti-rabbit, 1:5000. ZSGB-BIO, Beijing, China) for one hour at room temperature. To visualize the protein bands on the membranes, the SuperSignal West Pico Chemiluminescent Substrate (Thermo Fisher Scientific, MA, USA) was employed, and the Tanon 5200 chemiluminescent imaging system (Tanon, Shanghai, China) was used to capture images. For band density quantification, ImageJ software (NIH Image, Bethesda, MD, USA) was utilized, and protein expression was normalized to that of GAPDH. Supplementary Fig. S5 displays all uncropped blots.

### Statistical analysis

Valid data was obtained by independently repeating the experiments at least three times. The means ± SD were used to present the results. Statistical analysis and graphing were performed using the GraphPad Prism software (v8.0.1, La Jolla, CA, USA). To evaluate the significance between the two groups, a two-tailed unpaired Student’s t-test was conducted. Any *p*-values below 0.05 were considered significant.

## Results

### BBR promotes mouse primordial follicle activation in vitro

**Fig. 1:**
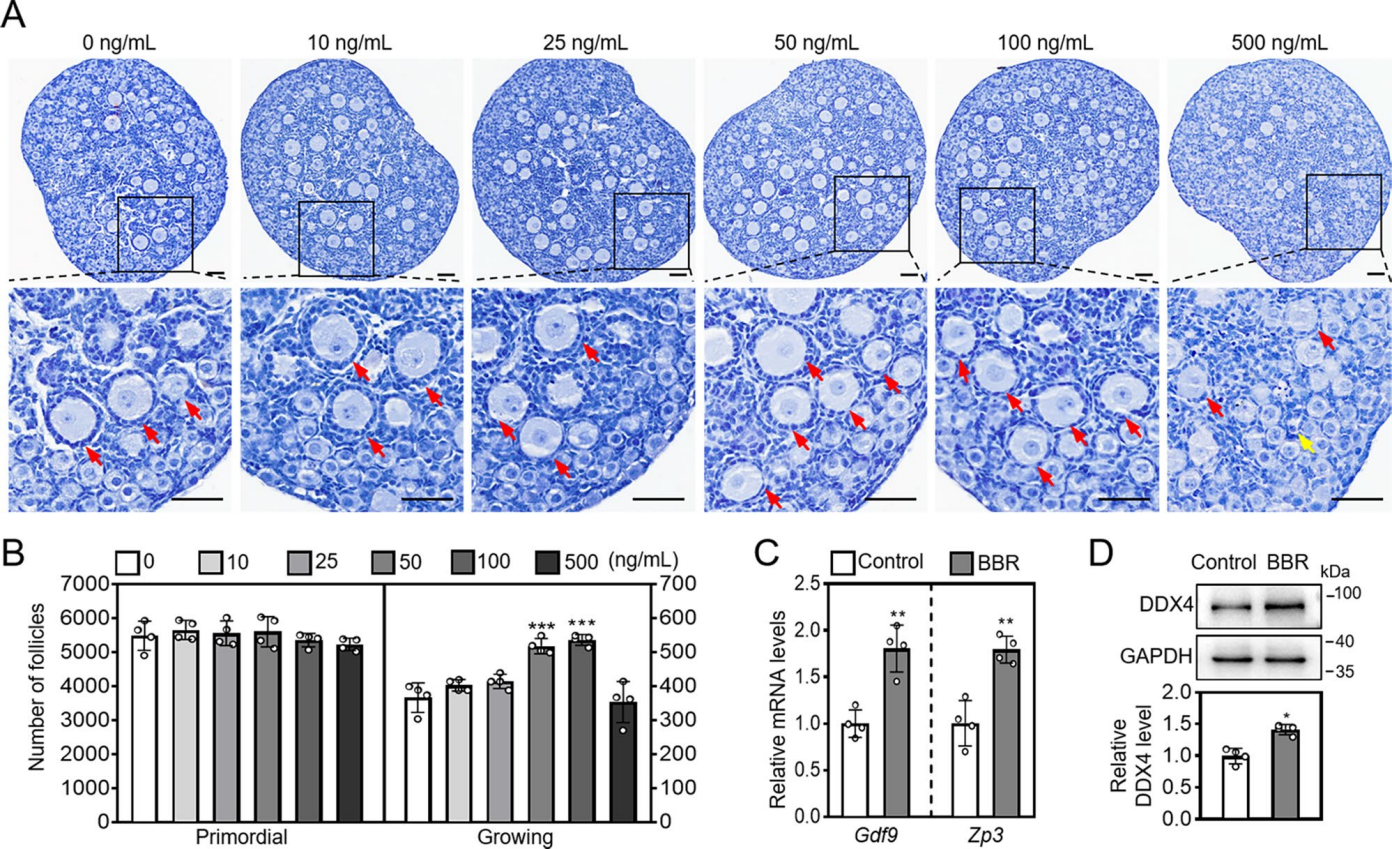
BBR promotes mouse primordial follicle activation in cultured mouse ovaries. Three dpp mouse ovaries were subjected to a four-day culture in the medium supplemented with 0-500 ng/mL BBR for follicle analysis, or a two-day culture in the medium without (control) or with 50 ng/mL BBR for gene and protein analysis. (**A** and **B**) The ovary morphological examination and primordial and growing follicle number. (**C** and **D**) The mRNA levels of *Gdf9* and *Zp3* and protein levels of DDX4. Hematoxylin dye was used to stain the nuclei. Red arrows, growing follicles; yellow arrows, atretic follicles. Scale bar = 50 μm. Data are presented as mean ± SD of four independent experiments. *p < 0.05, **p < 0.01, ***p < 0.001

In cultured neonatal mouse ovaries, 50 and 100 ng/mL BBR significantly increased growing follicle number (control: 367.5 ± 42.2; 50 ng/mL BBR: 518.75 ± 19.49; 100 ng/mL BBR: 537.50 ± 13.46. Figure 1A, B). However, 500 ng/mL BBR had no effect on growing follicle number, but significantly increased atretic follicle number (control: 37.50 ± 6.45; BBR: 68.75 ± 4.79, Supplementary Fig. S1A). Thus, we used 50 ng/mL BBR for the subsequent experiments. BBR also significantly increased zona pellucida 3 (*Zp3*) and growth differentiation factor 9 (*Gdf9*) mRNA levels, as well as DEAD-box helicase 4 (DDX4) protein levels (Fig. 1C, D).

**Fig. 2:**
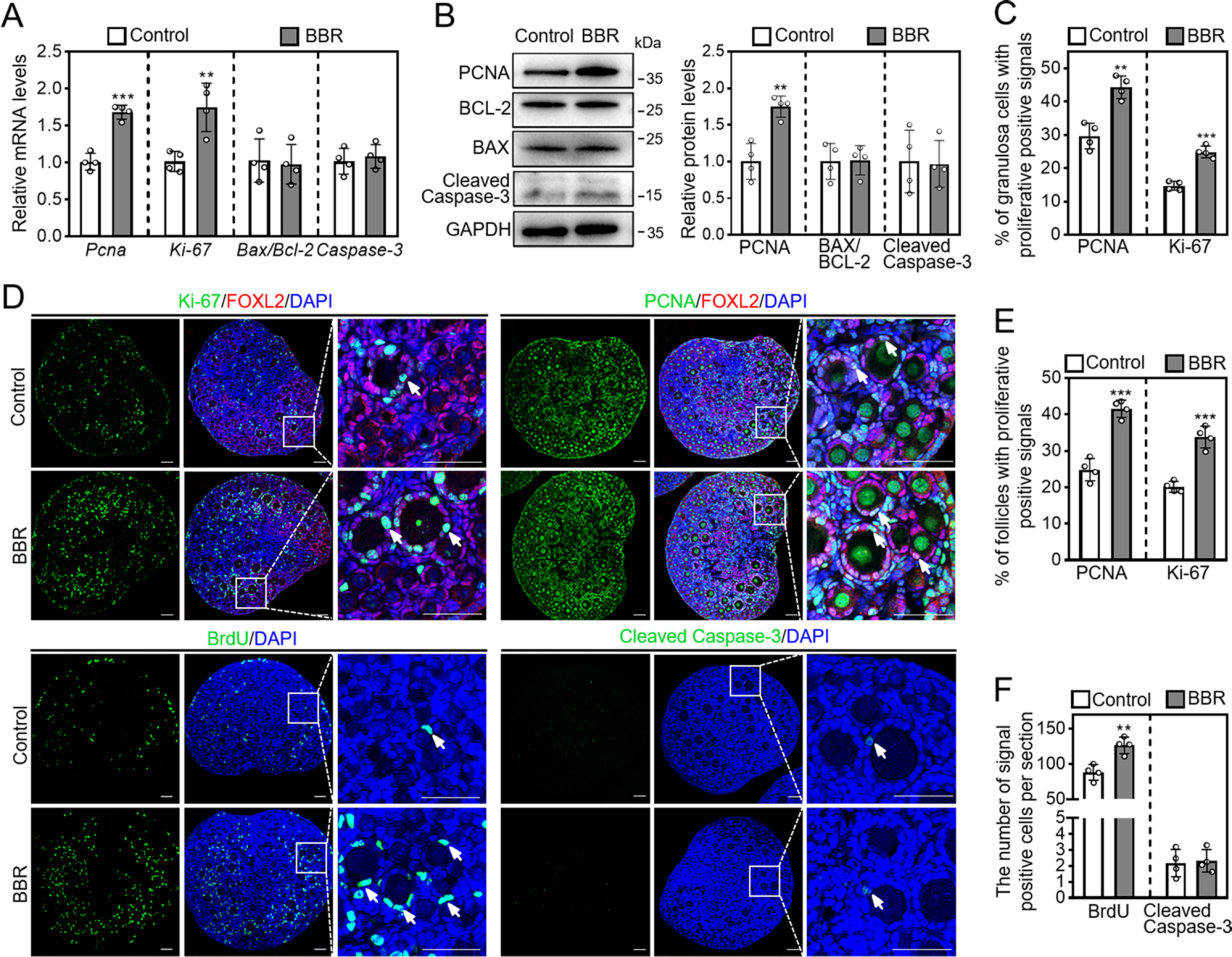
BBR promotes granulosa cell proliferation in cultured mouse ovaries. Three dpp mouse ovaries were subjected to a two-day culture in the medium without (control) or with 50 ng/mL BBR. (**A** and **B**) *Pcna*, *Ki-67*, *Bax/Bcl-2* and *Caspase-3* mRNA expressions and PCNA, BAX/BCL-2 and Cleaved Caspase-3 protein levels. (**C-F**) Ki-67, PCNA, BrdU, and Cleaved Caspase-3 immunofluorescence staining (green), and granulosa cells and follicles with positive signals proportion. Red, FOXL2; blue, DAPI. Arrows, signal-positive cells. Scale bar = 50 μm. Data are presented as mean ± SD of four independent experiments. **p < 0.01, ***p < 0.001

We further investigated whether BBR affected granulosa cell proliferation and apoptosis. BBR notably increased proliferating cell nuclear antigen (*Pcna*) and *Ki-67* mRNA levels and PCNA protein level (Fig. 2A, B). Nevertheless, BBR had no effect on the mRNA and protein levels of B-cell lymphoma 2-associated X (BAX)/ B-cell lymphoma 2 (BCL-2) or Cleaved Caspase-3, and the number of cells exhibiting Cleaved Caspase-3-positive signals (Fig. 2A, B, D, F). Consistent with these, BBR markedly increased PCNA- and Ki-67-positive granulosa cell percentage (PCNA: 29.60 ± 3.40% in control and 44.25 ± 2.98% in BBR; Ki-67: 14.70 ± 1.18% in control and 24.84 ± 1.56% in BBR), PCNA- and Ki-67-positive follicle percentage (PCNA: 24.81 ± 2.68% in control and 41.53 ± 2.05% in BBR; Ki-67: 20.07 ± 1.30% in control and 33.75 ± 2.63% in BBR), and BrdU-positive somatic cell number (control: 88.08 ± 9.70; BBR: 126.75 ± 10.14. Figure 2C-F). Thus, BBR promotes mouse primordial follicle activation *in vitro.*

### BBR activates primordial follicles via PI3K/Akt pathway in oocytes

**Fig. 3:**
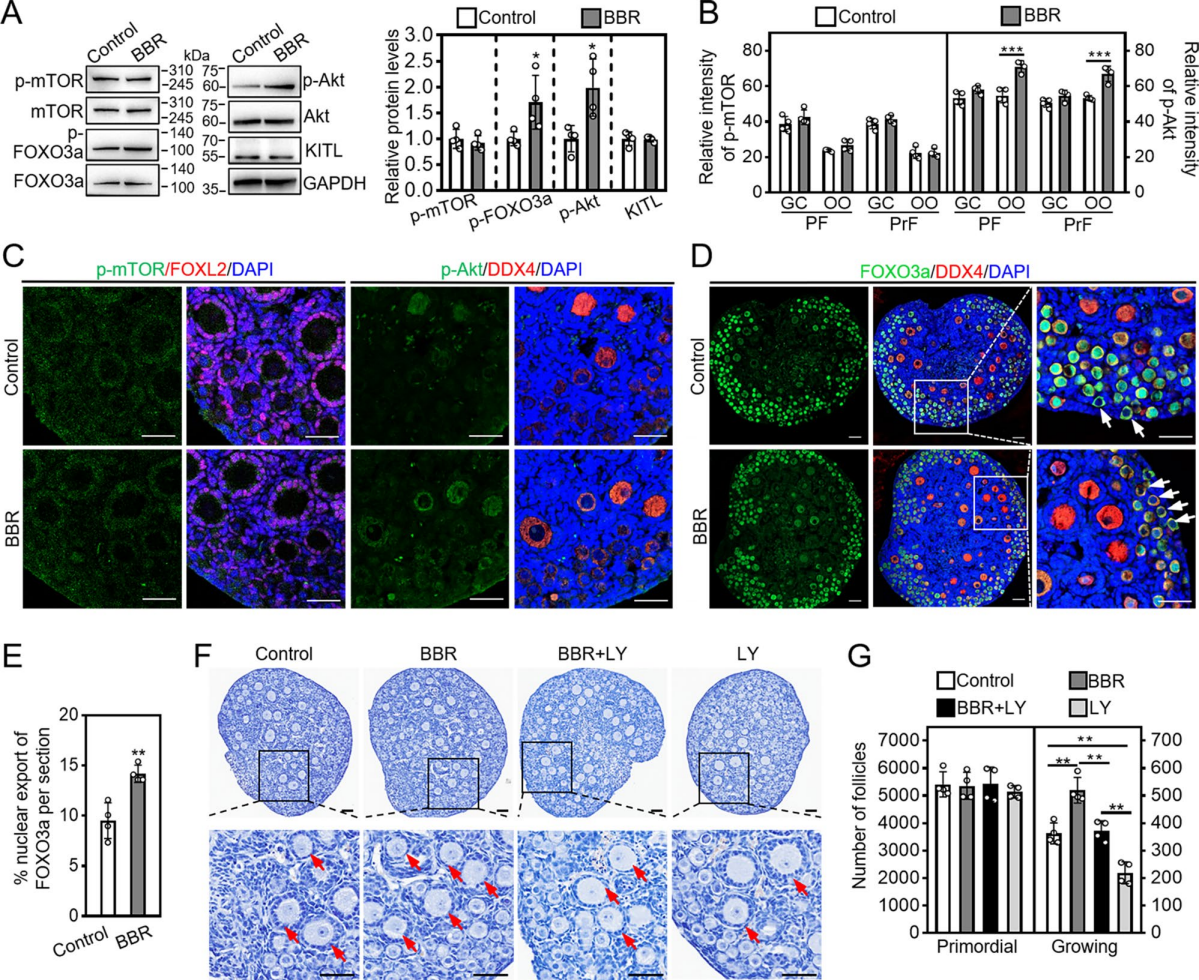
BBR activates PI3K/Akt pathway in mouse oocytes. Three dpp mouse ovaries were subjected to a one-day culture for the analysis of p-mTOR and p-Akt by western blotting and immunofluorescence staining, a two-day culture for the localization of FOXO3a by immunofluorescence staining, or a four-day culture for follicle analysis. (**A**) P-mTOR, p-FOXO3a, p-Akt and KITL protein levels. (**B** and **C**) Immunofluorescence staining (green) and the relative fluorescent intensities of p-mTOR and p-Akt in the granulosa cells (GC) and the oocytes (OO) of primordial follicles (PF) and primary follicles (PrF). (**D** and **E**) FOXO3a localization in oocytes cytoplasm (arrows) of primordial follicles and the proportion of oocytes displaying FOXO3a nuclear export. Red, FOXL2, DDX4, blue, DAPI. (**F** and **G**) The ovary morphology and primordial and growing follicle number. Hematoxylin dye was used to stain the nuclei. Red arrows, growing follicles. Scale bar = 50 μm. Data are presented as mean ± SD of four independent experiments. *p < 0.05, **p < 0.01, ***p < 0.001

In cultured neonatal moue ovaries, BBR significantly increased p-Akt and p-FOXO3a protein levels (Fig. 3A). Additionally, BBR significantly increased the relative fluorescence intensities of p-Akt in the primordial and primary follicle oocytes (Fig. 3B, C; Supplementary Fig. S1B). Using immunofluorescence staining, we found that BBR notably increased the percentage of oocytes that exhibited FOXO3a nuclear export (control: 8.75 ± 0.98%; BBR: 14.18 ± 0.74%. Figure 3D, E). The PI3K inhibitor LY294002 completely blocked BBR-promoted the number of growing follicles (control: 362.50 ± 37.97; BBR: 520.00 ± 40.77; BBR + LY: 372.50 ± 31.32. Figure 3F, G). Thus, BBR activates mouse primordial follicles through activating PI3K/Akt signaling within oocytes.

### BBR promotes the activation of mouse primordial follicles in vivo

**Fig. 4:**
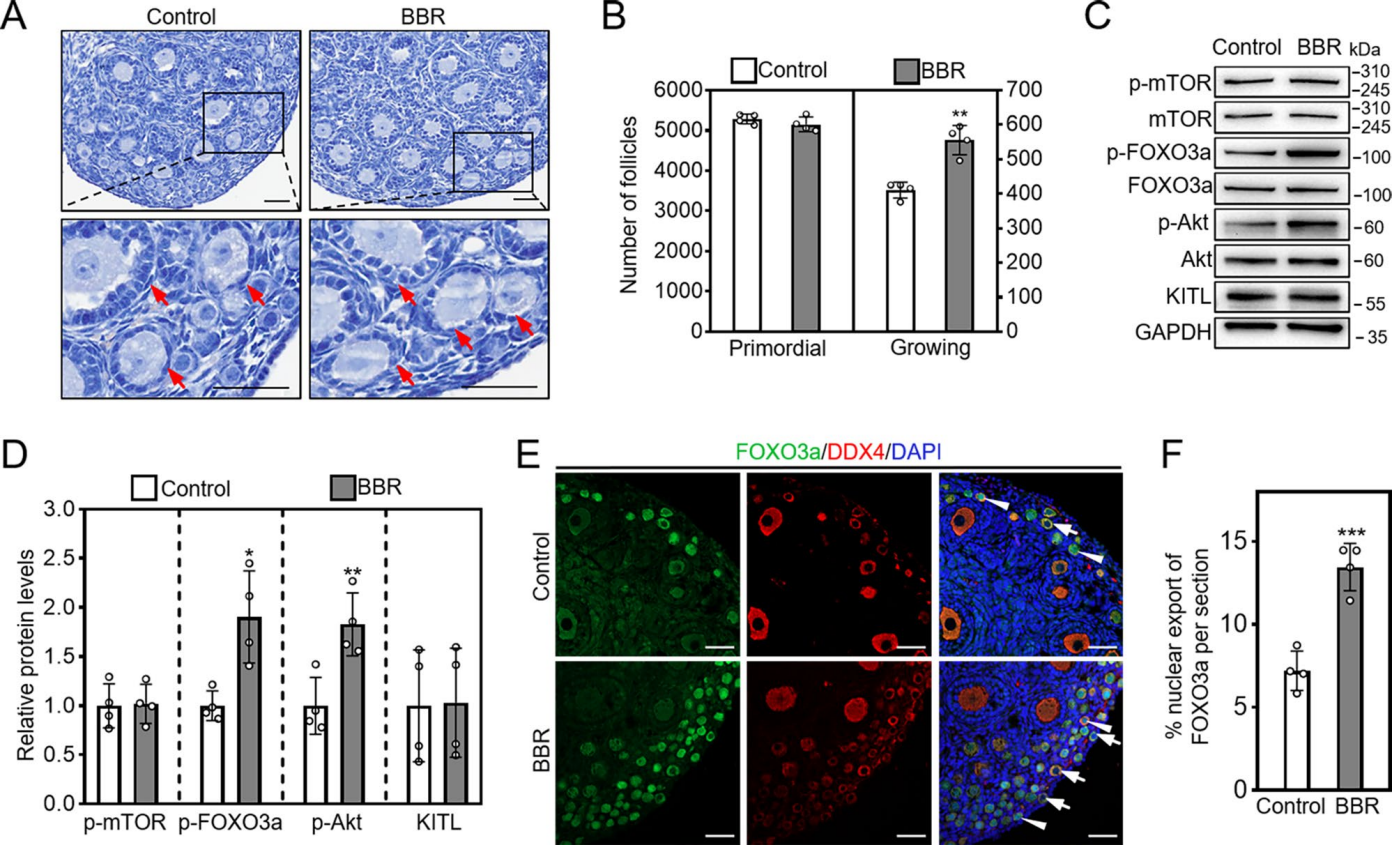
BBR promotes the activation of mouse primordial follicles in vivo. Three dpp female mice received daily intraperitoneally injection with physiological saline (Control) or 0.05 mg/kg BBR for two consecutive days. The ovaries were obtained 24 h after the end of injection for western blotting analysis and 48 h after the end of injection for follicle analysis and FOXO3a immunofluorescence staining. (**A** and **B**) Comparison of the ovary morphology and the number of primordial follicles and growing follicles. (**C** and **D**) P-mTOR, p-Akt, p-FOXO3a, and KITL protein levels. (**E** and **F**) FOXO3a localization in the nuclei (arrowheads) or cytoplasm (arrows) of primordial follicle oocytes, and the percentage of oocytes displaying FOXO3a nuclear export. Hematoxylin dye was used to stain the nuclei. Green, FOXO3a; red, DDX4; blue, DAPI. Red arrows, growing follicles. Scale bar = 50 μm. Data are presented as mean ± SD of four independent experiments. *p < 0.05, **p < 0.01, ***p < 0.001

**Fig. 5:**
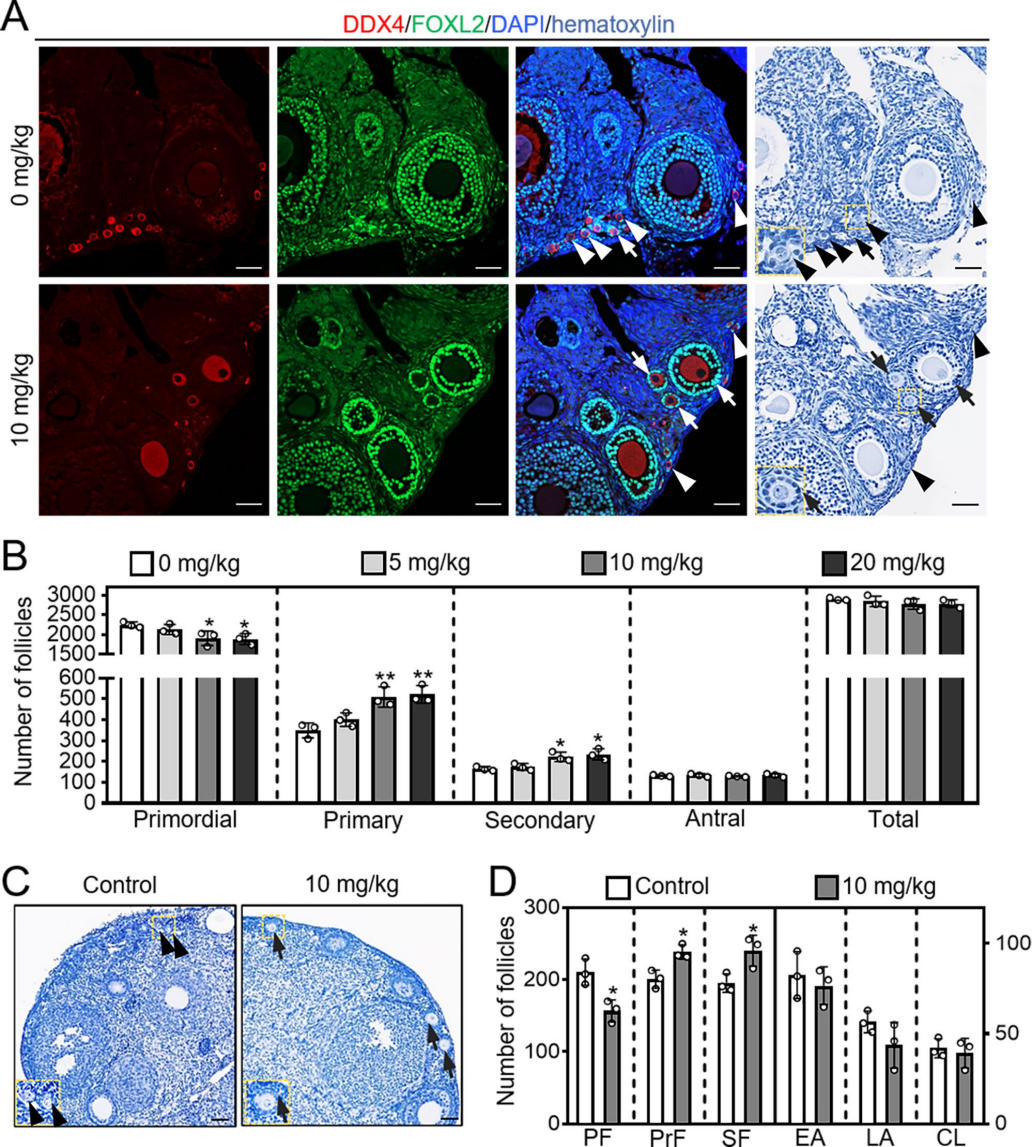
BBR intragastric administration promotes mouse primordial follicle activation. The adolescent and aged mice were intragastrically treated with BBR (0-20 mg/kg/day) for seven consecutive days, and the ovaries were collected at the end of intragastric administration for follicle counting. (**A** and **B**) Ovary morphology comparison and primordial and growing follicle number in adolescent mice. (**C** and **D**) Ovary morphology comparison and primordial and growing follicle number in aged mice. The image of the enlarged areas is indicated by the small yellow boxes, as displayed in the lower left corners. Hematoxylin dye was used to stain the nuclei. Green, FOXL2; red, DDX4; blue, DAPI. PF, primordial follicles; PrF, primary follicles; SF, secondary follicles; EA, early antral follicles; LA, late antral follicles; CL, corpus luteum. Arrowheads, primordial follicles; arrows, growing follicles. Scale bar = 50 μm. Data are presented as mean ± SD of three independent experiments. *p < 0.05, **p < 0.01

In neonatal mouse intraperitoneal injection experiment, BBR markedly increased growing follicle number (control: 410.00 ± 20.62; BBR: 555.00 ± 37.42. Figure 4A, B). BBR also significantly increased p-Akt and p-FOXO3a protein levels, and the percentage of oocytes exhibiting FOXO3a nuclear export (control: 7.213 ± 1.03%; BBR: 13.47 ± 1.43%. Figure 4C-F). Furthermore, the adolescent mice were intragastrically administered gradient concentrations of BBR for one week, and 10 mg/kg/day BBR significantly increased the number of primary follicles (0 mg/kg: 350.00 ± 28.58; 10 mg/kg: 510.00 ± 39.37) and secondary follicles (0 mg/kg: 165.70 ± 8.96; 10 mg/kg: 223.30 ± 17.61), but showed no obvious effects on the morphologies of the ovaries, vital organs and mouse body weight (Fig. 5A, B; Supplementary Fig. S2A, B; Fig. S3A-C). Thus, we used 10 mg/kg/day BBR for the subsequent experiments. Next, the aged mice were intragastrically administered BBR. BBR significantly increased the number of primary follicles (control: 201.00 ± 10.23; 10 mg/kg: 238.70 ± 8.06) and secondary follicles (control: 195.70 ± 10.34; 10 mg/kg: 240.30 ± 21.36. Figure 5C, D; Supplementary Fig. S2C). Thus, BBR promotes the activation of mouse primordial follicles activation in vivo.

### BBR intragastric administration improves the quantity of ovulated oocyte in aged mice

**Fig. 6:**
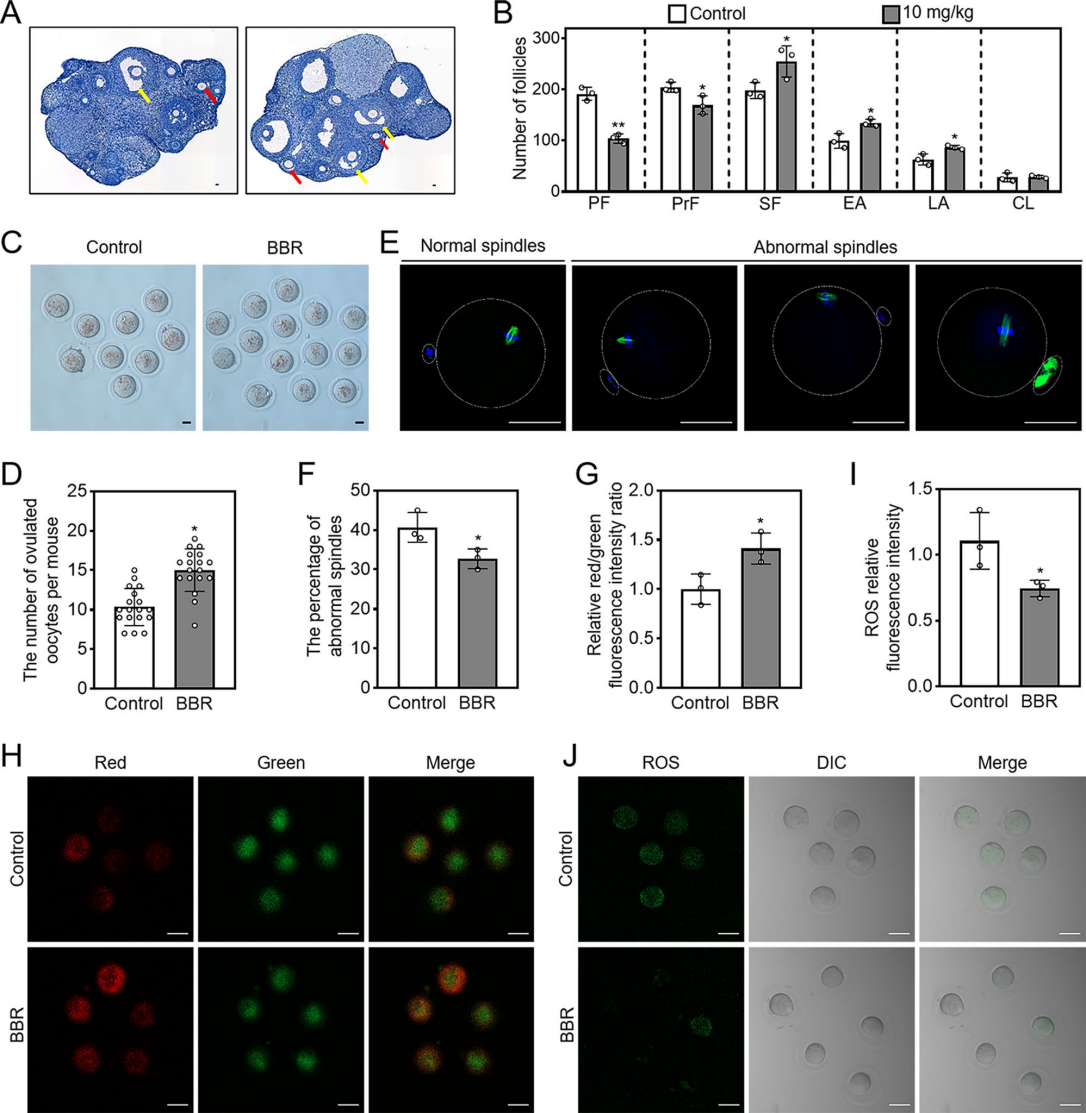
BBR increases the quantity of ovulated oocyte in aged mice. The aged mice were intragastrically treated with BBR (10 mg/kg/day) for seven days, followed by a three-week routine feeding period. The ovaries were then collected for follicle counting and oocytes were obtained for quantity and quality analysis. (**A** and **B**) Ovary morphology comparison and primordial and growing follicle number. (**C** and **D**) Comparison and the number of ovulated oocytes. (**E** and **F**) Immunofluorescence staining of normal and abnormal spindles and the percentage of abnormal spindles. (**G** and **H**) Ratio of relative red/green fluorescence intensity and oocyte ΔΨm as shown by JC-1 staining. (**I** and **J**) The relative fluorescence intensity and fluorescence staining (green) of ROS. Hematoxylin dye was used to stain the nuclei. Red arrows. EA; yellow arrows, LA. Scale bar = 50 μm. Data are presented as mean ± SD of three independent experiments. *p < 0.05

The female aged mice were intragastrically administered BBR for one week, followed by a three-week routine feeding. BBR significantly increased the number of secondary follicles (control: 198.00 ± 12.96; 10 mg/kg: 255.00 ± 25.02), early antral follicles (control: 99.67 ± 11.90; 10 mg/kg: 134.70 ± 6.02) and late antral follicles (control: 63.00 ± 8.98; 10 mg/kg: 86.67 ± 3.30), but did not affect the number of corpus luteum or the mice body weight (Fig. 6A, B; Supplementary Fig. S4A-C). BBR also notably enhanced ovulated oocytes number (control: 10.33 ± 2.29; BBR: 15.00 ± 2.62, *n* = 18) and the value of oocyte ΔΨm, but significantly decreased abnormal spindle percentage (control: 40.67 ± 3.09%; BBR: 32.67 ± 2.05%) and the content of ROS (Fig. 6C-J). The findings demonstrate that BBR intragastric administration leads to an increase in the quantity and the potential quality of ovulated oocytes in aged mice.

**Fig. 7:**
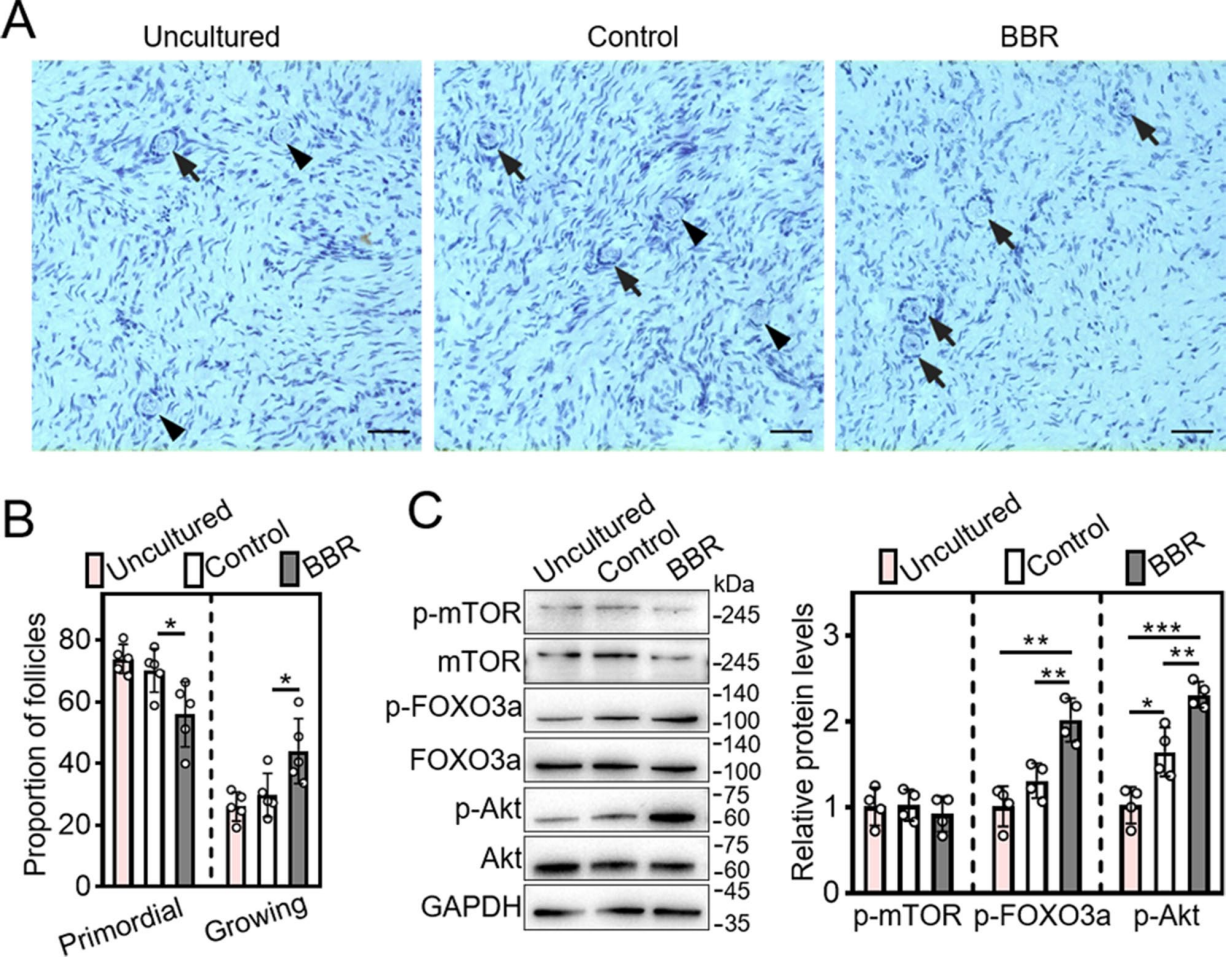
BBR promotes human primordial follicle activation in vitro. Human ovarian tissue fragments were collected directly (uncultured) or subjected to a four-day culture in medium either without (control) or with 50 ng/mL BBR, followed by a two-day culture in medium. The tissues were collected after four days for western blotting analysis or after six days for follicle counting. (**A** and **B**) Human ovarian tissue morphology comparison and primordial and growing follicle percentage. (**C**) P-mTOR, p-Akt and p-FOXO3a protein levels. Hematoxylin dye was used to stain the nuclei. Arrowheads, primordial follicles; arrows, growing follicles. Scale bar = 50 μm. Data are presented as mean ± SD of at least four independent experiments. *p < 0.05, **p < 0.01, ***p < 0.001

**Fig. 8:**
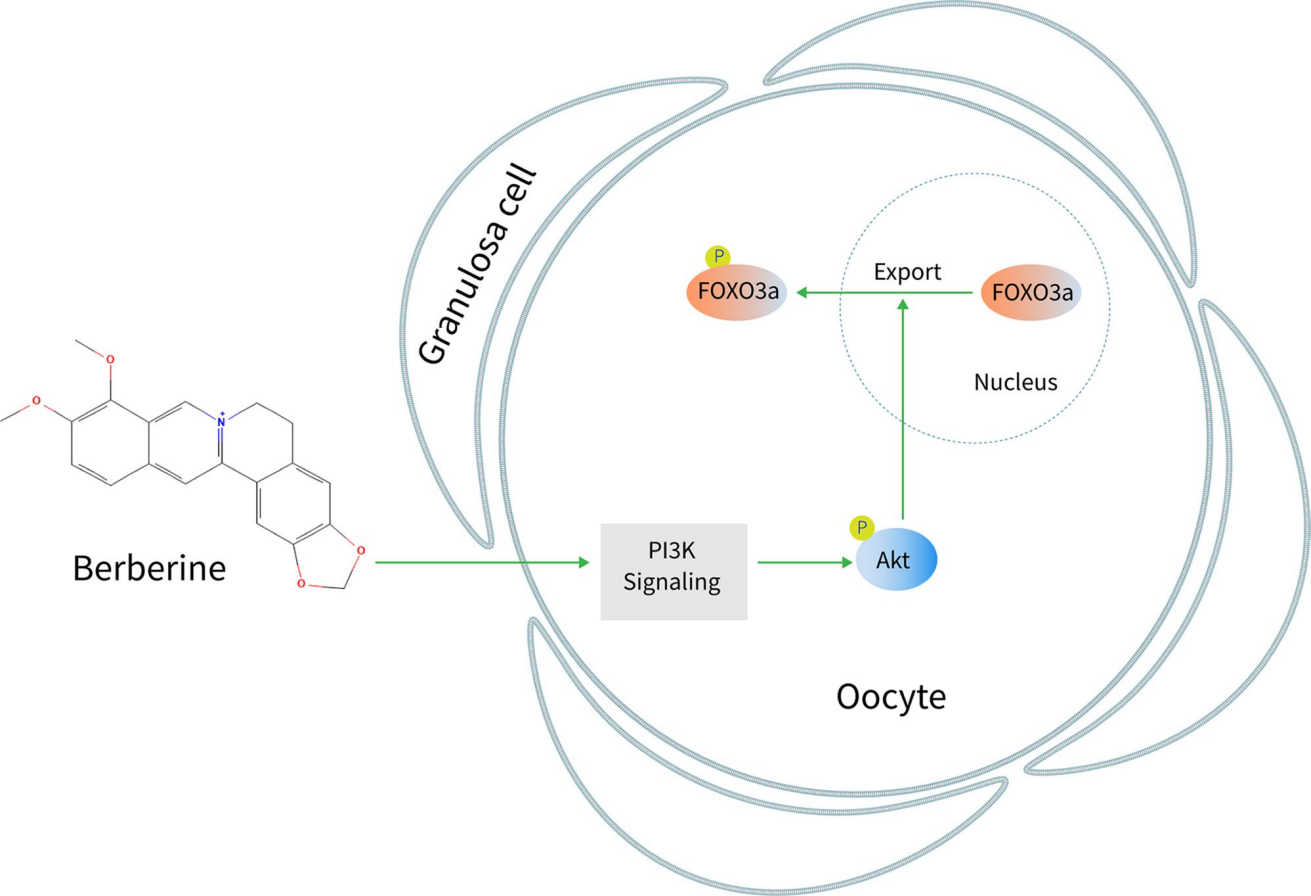
A schematic illustrating primordial follicle activation by BBR. BBR enters oocytes of primordial follicles, and then activates the PI3K/Akt signaling, resulting in primordial follicle activation

### BBR promotes human primordial follicle activation in vitro

In cultured human ovarian fragments, BBR significantly increased growing follicle proportion (control: 29.79 ± 6.95%; BBR: 44.06 ± 9.40%) as well as p-Akt and p-FOXO3a protein levels compared with those of the control (Fig. 7A-C). Thus, BBR could promote human primordial follicle activation by activating PI3K/Akt signaling. Otherwise, growing follicle proportion increased slightly but p-Akt protein levels increased significantly compared with those of the uncultured group, suggesting that the primordial follicles are initially growing during the culture (Fig. 7A-C).

## Discussion

BBR is an effective oral medicine for treating intestinal infections caused by bacteria and virus (Yu et al. 2020; Zhang et al. 2021a). In our study, BBR promoted the activation of mouse and human primordial follicles via the PI3K/Akt pathway. Moreover, BBR intragastric administration increased the quantity of ovulated oocytes in aged mice.

BBR promoted the activation of mouse primordial follicles in vitro and in vivo and human primordial follicles by ovarian tissue culture, suggesting that BBR could promote the activation of primordial follicles in mammals. The PI3K/Akt pathway in oocytes plays a crucial role in primordial follicle activation (Maidarti et al. 2020; Zhang et al. 2024b). BBR could enter into the cytoplasm of CCD-18Co cells (Guan et al. 2018), increase p-Akt levels in the ovaries of letrozole-induced PCOS rats (Zhang et al. 2020b) and in the heart of LPS-induced septic mice (Zhang et al. 2022a). It has also been reported that BBR activates the PI3K/Akt signaling pathway in cultured rat granulosa cells (Yu et al. 2021). Consistent with these, BBR increased p-Akt levels in mouse and human ovarian tissues during primordial follicle activation, and increased the immunofluorescence intensities of p-Akt in oocytes of cultured mouse ovaries. BBR-promoted mouse primordial follicle activation was completely blocked by the PI3K inhibitor LY294002. Thus, BBR enters into the oocytes and activates the PI3K/Akt pathway, resulting in primordial follicle activation.

The aged mice were used as a low-fertility model to evaluate the effectiveness of medicines (Jiao et al. 2022; Han et al. 2023). In our study, BBR intragastric administration promoted primordial follicle activation, and increased the quantity of ovulated oocytes in aged mice without observing toxicity or adverse effects. The increase in the number of ovulated oocytes could possibly be attributed to the enhancement of primordial follicle activation. We also found that BBR increased the levels of ΔΨm and decreased the content of ROS and the percentage of abnormal spindles, suggesting that BBR improves oocyte quality. These are consistent with previous studies that BBR decreases intracellular ROS content and improves mitochondrial function in PC-12 cells, ARPE-19 cells, and the retinal pigment epithelium of aged mice (Chen et al. 2022; Yuan et al. 2023). Thus, BBR improves the quantity and potential quality of ovulated oocytes in aged mice, by which BBR may rescue infertility in aged mice.

Patients with POI have a few of residual primordial follicles that cannot be activated physically (Chon et al. 2021). Women of advanced age exhibit decreasing ovarian reserve, accompanied by a decrease in oocyte developmental competence (Zhang et al. 2020a; Smits et al. 2021). All of these ultimately lead to reduced fertility. Current research on the therapy of POI mainly includes IVA, PRP, and stem cells (Huang et al. 2022). However, these therapies are limited to individual cases, and are not widely applied in clinical practice due to the invasive nature of the procedures and their low success rate (Ding et al. 2023). Here, we demonstrated that the intragastric administration of BBR could increase the quantity and potential quality of ovulated oocyte in aged mice. The dosage of BBR in aged mice was 10 mg/kg/day. BBR at 50 mg/kg and 95 mg/kg/day is used to treat rat atherosclerosis and mouse PCOS, respectively (Shi et al. 2018; Yu et al. 2021). BBR at 14 mg/kg/day was used to treat patients with PCOS (Mishra et al. 2022). Thus, BBR, a safe oral medication, may be a candidate for treating POI patients and aged women.

In summary, BBR activated primordial follicles of mouse and human via oocyte PI3K/Akt pathway (Fig. 8), and increased the quantity of ovulated oocytes in aged mice. As a safe oral medication, BBR may be a potential treatment for rescuing infertility in POI patients and aged women.

## Electronic Supplementary Material

Below is the link to the electronic supplementary material.

Supplementary Material 1

## Data Availability

The datasets used and/or analyzed during the current study are available from the corresponding author on reasonable request.

## Abbreviations

Akt: Protein kinase B
ART: Assisted reproductive technology
Bax: B-cell lymphoma 2-associated X
BBR: Berberine
Bcl-2: B-cell lymphoma 2
BrdU: Bromodeoxyuridine
CL: Corpus luteum
COCs: Cumulus-oocyte complexes
DAPI: 4’,6-diamidino-2-phenylindole
DDX4: DEAD-box helicase 4
DMSO: Dimethyl sulfoxide
dpp: Day postpartum
EA: Early antral follicles
FOXO3a: Forkhead Box O3a
GC: Granulosa cells
Gdf9: Growth differentiation factor 9
IVA: In vitro activation
KITL: Proto-oncogenic receptor tyrosine kinase ligand
LA: Late antral follicles
LPS: Lipopolysaccharide
mTOR: Mammalian target of rapamycin
NF: Nuclear factor
OO: Oocytes
PBS: Phosphate-buffered saline
PCNA: Proliferating cell nuclear antigen
PCOS: Polycystic ovary syndrome
PF: Primordial follicle
PFA: Paraformaldehyde
PI3K: Phosphatidylinositol 3-kinase
PMSG: Pregnant mare serum gonadotropin
POI: Premature ovarian insufficiency
PrF: Primary follicles
PRP: Platelet-rich plasma
PVDF: Polyvinylidene fluoride
qRT-PCR: Quantitative real-time PCR
ROS: Reactive oxygen species
Rpl19: Ribosomal protein L19
SF: Secondary follicles
SDS: Sodium dodecyl sulfate
Zp3: Zona pellucida 3
ΔΨm: Mitochondrial membrane potential
