# Supplementary material for: Berberine promotes primordial follicle activation and increases ovulated oocyte quantity in aged mice
Source: Mol Med. 2024 Dec 20;30:251. doi: 10.1186/s10020-024-01042-z (PMC11660874; doi:10.1186/s10020-024-01042-z)
Supplement: Supplementary file 1 — Supplementary Material 1 [file 10020_2024_1042_MOESM1_ESM.docx]

**Berberine promotes primordial follicle activation and** **increases ovulated oocyte quantity in aged mice**

Shuang Liu^1^, Weiyong Wang^1^, Huiyu Liu^1^, Hongwei Wei^1^, Yashuang Weng^1^, Wenjun Zhou^1^, Xiaodan Zhang^1^, Sihui He^1^, Ye Chen^1^, Yahong Wang^2^, Meijia Zhang^1,*^, Xin Chen^2,*^

^1^The Innovation Centre of Ministry of Education for Development and Diseases, School of Medicine, South China University of Technology, Guangzhou 510006, China

^2^Reproductive Medicine Center, Shunde Hospital of Southern Medical University (The First People’s Hospital of Shunde), Foshan 528300, China

*Corresponding author: Meijia Zhang, The Innovation Centre of Ministry of Education for Development and Diseases, School of Medicine, South China University of Technology, 382 Outer Ring East Road, Guangzhou Higher Education Mega Center, Guangzhou 510006, Guangdong, China. E-mail: [zhangmeijia@scut.edu.cn](mailto:zhangmeijia@scut.edu.cn);

Xin Chen, Reproductive Medicine Center, Shunde Hospital of Southern Medical University (The First People’s Hospital of Shunde), 1 Jiazi Road, Foshan 528300, Guangdong, China. E-mail: [chenxin4672@smu.edu.cn](mailto:chenxin4672@smu.edu.cn).


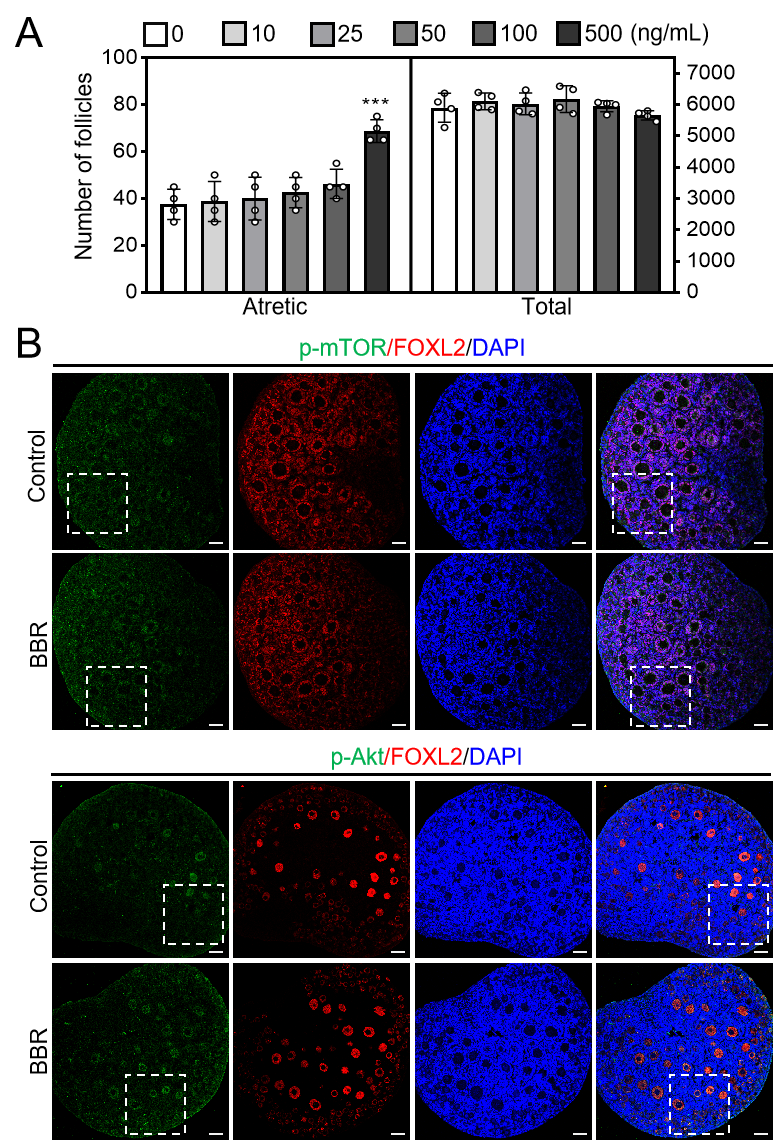


**Supplementary Fig S1.** **BBR promotes mouse primordial follicle activation *in vitro*.** The ovaries from 3 dpp mice were cultured in the medium supplemented with gradient concentrations of BBR (0-500 ng/mL) for 4 days (A), or cultured in the medium without (control) or with BBR (50 ng/mL ) for 1 day (B). (A) The number of atretic and total follicles in the different groups. (B) Immunofluorescence staining of p-mTOR and p-Akt (green) in the two groups. FOXL2, red; DAPI, blue. The amplified views of the boxed area are shown in Fig 3C. Scale bars = 50 µm. Bars indicate the mean ± SD. ****p* < 0.001.


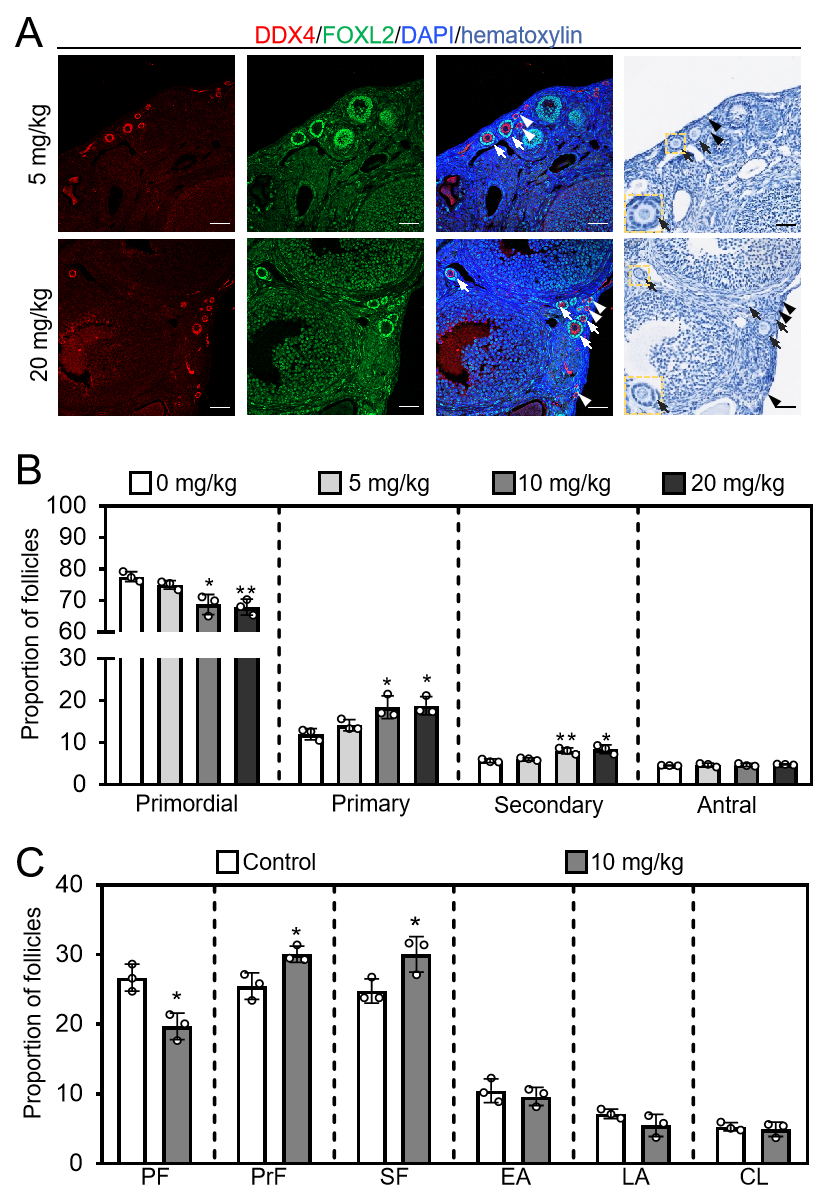


**Supplementary Fig S2.** **Effects of BBR intragastric administration on primordial follicle activation.** The adolescent and aged mice were treated intragastrically with BBR (0-20 mg/kg/day, A-C) for one week. (A) Immunofluorescence staining and morphological comparison of ovaries in different groups of adolescent mice. (B) The proportion of primordial and growing follicles including primary, secondary and antral follicles in the different groups of adolescent mice. (C) The proportion of primordial (PF), primary (PrF), secondary (SF), early antral (EA) and late antral follicles (LA), and corpus luteum (CL) in the two groups of aged mice. All the experiments were independently repeated three times. Scale bars = 50 µm. Bars indicate the mean ± SD. **p* < 0.05, ***p*< 0.01.


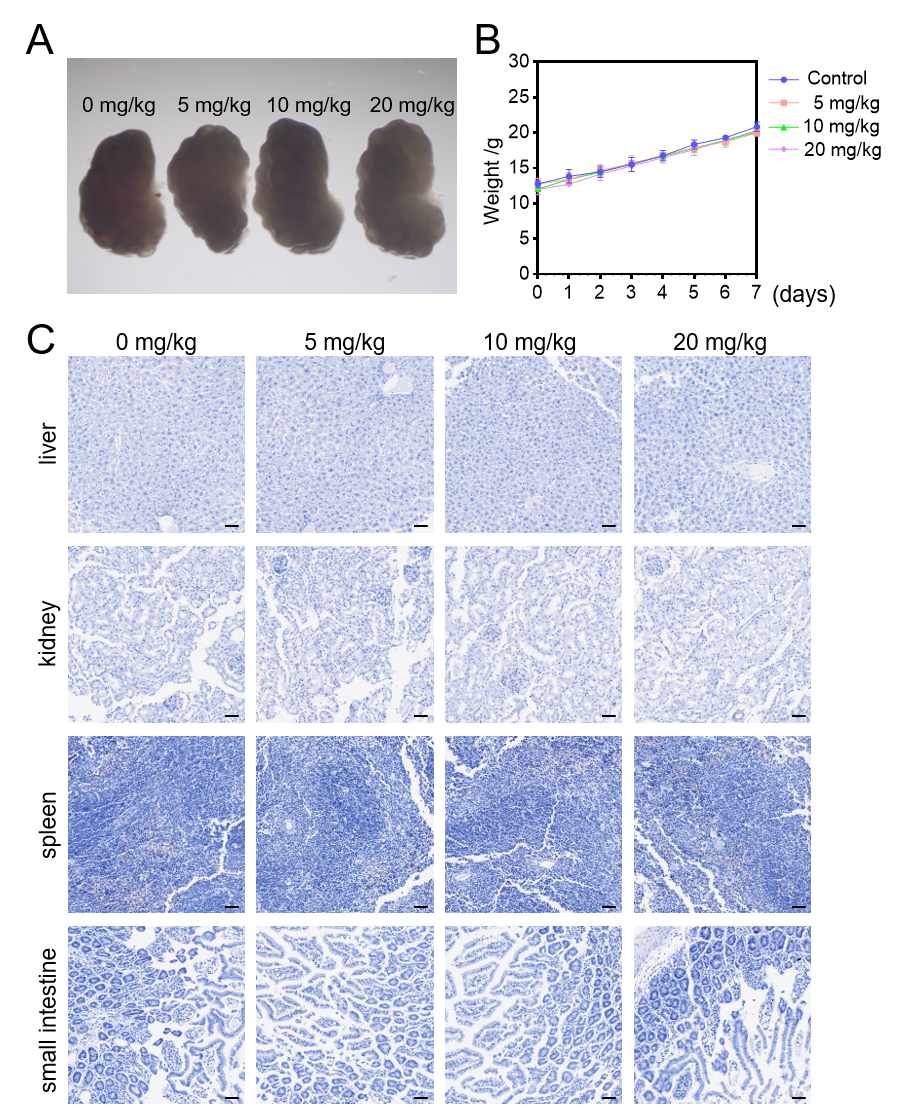


**Supplementary Fig S3.** **Effects of BBR intragastric administration on body weight and organs in the adolescent mice.** The adolescent mice were treated intragastrically with BBR (0-20 mg/kg/day) for one week, and the organs were collected at the end of intragastric administration. (A) The ovarian morphologies in the different groups. (B) The body weight change in the different groups. (C) Morphological comparison of liver, kidney, spleen, and small intestine in the different groups. Scale bars = 50 µm. Bars indicate the mean ± SD.


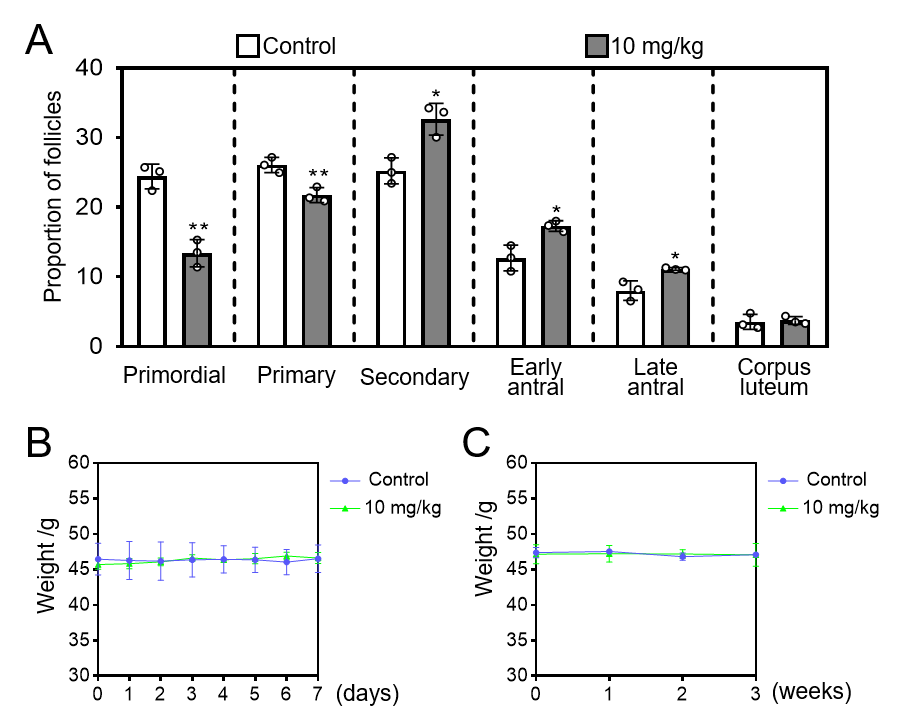


**Supplementary Fig S4.** **Effects of BBR intragastric administration on primordial follicle activation and body weight in the aged mice.** The aged mice were treated intragastrically with BBR (10 mg/kg/day) for one week, and then fed for further 3 weeks. (A) The proportion of primordial, primary, secondary, early antral and late antral follicles, and corpora lutea in the two groups. (B-C) The body weight change in the two groups. All the experiments were independently repeated three times. Bars indicate the mean ± SD. **p* < 0.05, ***p*< 0.01.


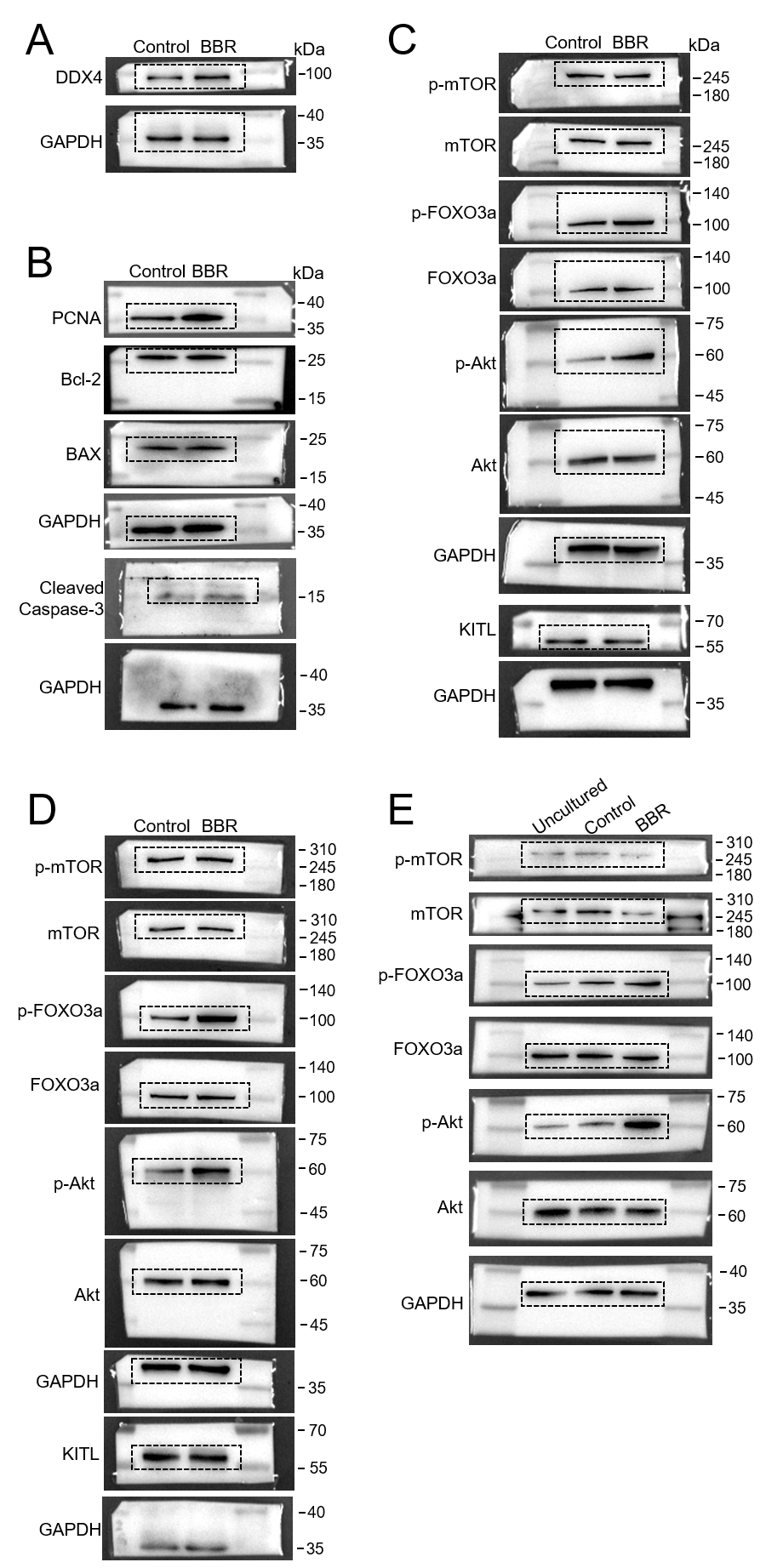


**Supplementary Fig S5.** **Uncropped scans of the western blotting results in Fig 1, 2, 3, 4 and 7.** The blots in the black dashed line boxes were used in Fig 1D (A)**,** 2B (B)**,** 3A (C)**,** 4C (D)**,** and 7C (E).

**Supplementary Table S1.** List of primary antibodies used in immune detection in this study.

| Antibody | Catalog  Code | Source | Host | Dilution | |
| --- | --- | --- | --- | --- | --- |
|  |  |  |  | IF | WB |
| Akt | 4691 | Cell Signaling Technology | Rabbit | — | 1:1000 |
| p-Akt | 4060 | Cell Signaling Technology | Rabbit | 1:200 | 1:1000 |
| BrdU | ab1893 | Abcam | Sheep | 1:200 | — |
| BAX | 50599-2-Ig | Proteintech | Rabbit | — | 1:1000 |
| BCL2 | 26593- 1-AP | Proteintech | Rabbit | — | 1:1000 |
| Cleaved  Caspase-3 | 9664 | Cell Signaling Technology | Rabbit | 1:50 | 1:1000 |
| DDX4 | ab27591 | Abcam | Mouse | 1:200 | 1:1000 |
| FOXL2 | NB100-1277 | Novus Biologicals | Goat | 1:300 | 1:1000 |
| FOXO3a | 12829 | Cell Signaling Technology | Rabbit | 1:200 | 1:1000 |
| p-FOXO3a | ab26649 | Abcam | Rabbit | — | 1:1000 |
| Ki-67 | 9129s | Cell Signaling Technology | Rabbit | 1:300 | — |
| KITL | sc-13126 | Santa Cruz Biotechnology | Mouse | — | 1:1000 |
| mTOR | 2972 | Cell Signaling Technology | Rabbit | — | 1:1000 |
| p-mTOR | 2971 | Cell Signaling Technology | Rabbit | 1:100 | 1:1000 |
| PCNA | 2586 | Cell Signaling Technology | Rabbit | 1:300 | 1:1000 |
| Alexa Fluor^®^ 488 α-Tubulin | ab197737 | Abcam | Rabbit | 1:400 | — |
| GAPDH | 2118 | Cell Signaling Technology | Rabbit | — | 1:1000 |

IF: Immunofluorescence; WB: Western blotting

**Supplementary Table S2**. Primers for qRT‒PCR used in this study.

| Genes | Forwards (5’-3’) | Backwards (5’-3’) |
| --- | --- | --- |
| *Bax* | TTTCATCCAGGATCGAGCAGG | GCAAAGTAGAAGAGGGCAACCAC |
| *Bcl2* | CTACCGTCGTGACTTCGCA | TACCCAGCCTCCGTTATCC |
| *Caspase-3* | CCGGTTACTATTCCTGGAGA | TAACACGAGTGAGGATGTGC |
| *Gdf9* | TCTTAGTAGCCTTAGCTCTCAGG | TGTCAGTCCCATCTACAGGCA |
| *Ki-67* | ATCATTGACCGCTCCTTTAGGT | GCTCGCCTTGATGGTTCCT |
| *Pcna* | CGGCGTGAACCTGCAGAGCA | GGTTGCGGTCGCAGCGGTAT |
| *Rpl19* | CTGAAGGTCAAAGGGAATGTGTTC | TGGTCAGCCAGGAGCTTCTTG |
| *Zp3* | CCTCAGGACTAACCGTGTGGA | CCATCAGGCGAAGAGAGAAAG |
